# The Long-Term Clinical and Radiographic Outcomes of Cerclage Cable Fixation for Displaced Acetabular Fractures Using a Posterior Approach: A Retrospective Cohort Study

**DOI:** 10.3390/medicina60101659

**Published:** 2024-10-10

**Authors:** Yutaro Kuwahara, Genta Takemoto, So Mitsuya, Ken-ichi Yamauchi

**Affiliations:** Department of Orthopedic Surgery, Toyohashi Municipal Hospital, Toyohashi 441-8570, Aichi, Japan; pauro-genta-3sey-megumi@hotmail.co.jp (G.T.); trisagittalis@gmail.com (S.M.); yamauchi-kenichi@toyohashi-mh.jp (K.-i.Y.)

**Keywords:** acetabulum fracture, cerclage cable fixation, complication, long-term follow-up

## Abstract

*Background and Objectives*: Cerclage cable fixation with 2 mm multiple-braided cables for displaced acetabular fractures has shown good midterm functional and radiographic outcomes. We retrospectively evaluated the clinical and radiographic outcomes of cerclage cable fixations over ten years. *Materials and Methods*: We extracted data for patients who underwent cerclage cable fixation for acetabular fractures at a single institution from 2007 to 2012. We adopted this procedure for acetabulum fractures with posterior column fractures. Postoperative reduction quality, complications, reoperations, and Japanese Orthopedic Association (JOA) hip objective functional scores were analyzed. Postoperative reduction quality was classified using plain radiography and computed tomography. *Results*: We evaluated nine patients with a mean follow-up period of 14.1 ± 2.6 years (range: 10.8–18.1 years). The mean age was 47.1 ± 15.5 years old (range: 28–74 years); the mean injury severity score was 13.6 ± 4.7 (range: 9–22). The most frequent type of fracture was a both-column fracture. Anatomical reduction quality was achieved in five cases. Four patients had hip osteoarthritis at the last follow-up; among them, one patient had worsening hip arthritis > 5 years after surgery, and one patient developed osteoarthritis > 10 years after surgery. Their postoperative reduction quality was worse than their anatomical reduction quality, and both engaged in physical labor. None of the patients underwent revision total hip arthroplasty. The mean JOA hip score was 90.9 ± 7.9 (range: 74–100); seven patients scored >90 at the last follow-up. *Conclusions*: Cerclage cable fixation showed satisfactory postoperative reductions and favorable long-term clinical outcomes. Long-term follow-up might be necessary for patients whose postoperative reduction is not anatomical to detect late occurrence of hip osteoarthritis, even if osteoarthritis is not evident during short-term follow-up periods.

## 1. Introduction

Acetabular fractures are uncommon injuries, with a reported incidence rate of 3 per 100,000 population per year [1]. In 1964, Judet and Letournel first reported that displaced acetabular fractures have better outcomes from open reduction and internal fixation than from conservative treatment [2]. However, these fractures have various patterns and are difficult to restore anatomically. Therefore, many approaches and fixation methods have been reported [3].

Plate fixation via an anterior approach, such as an ilioinguinal approach, is used most widely for acetabulum fractures, including anterior column fractures, and has shown good functional and radiographic results [4,5]. These approaches do not allow for direct viewing of the articular surface, and intraoperative hardware insertion into the articular surface has been reported [3]. Plate fixation via a posterior approach is also adopted for acetabulum fractures such as posterior column and posterior wall fractures. These procedures provide a direct view of the articular surfaces for reduction. However, reducing the quadrilateral surface (the medial component of the hip joint) would be difficult. Combining both approaches is ideal for anatomical reduction, and good postoperative clinical outcomes have been reported; however, it is more invasive [6]. Percutaneous screw fixation with a navigation system has recently been reported. These methods enable precise screw insertion but have the disadvantage that they do not reduce the articular surfaces and are not available at all institutions [7,8]. The incidence rate of total hip arthroplasty (THA) revisions after fixation for acetabulum fractures is reportedly 10.5% [3]. In THA procedures, screws inserted into the subchondral bone for supporting the articular surface may interfere with acetabular cup placement and need to be removed before the acetabular surface is reamed, causing longer operative times and greater blood loss. Cerclage cable fixation with 2 mm multiple-braided cables via a posterior approach was first introduced by Kang et al. in 2002 [9]. This surgical technique fixes the fracture by fastening the lateral and medial component of the acetabulum above the acetabular roof and providing indirect reduction and fixation of large quadrilateral surface fragments without subchondral screw insertion via a posterior-only approach.

Since 2007, we have been using cerclage cable fixation for acetabular fractures, including posterior column articular fractures, via the posterior approach. This study retrospectively evaluated the clinical and radiographic outcomes of cerclage cable fixation over ten years.

## 2. Materials and Methods

### 2.1. Participants

In this retrospective cohort study, we collected data for patients who underwent cerclage cable fixation for acetabular fractures at our hospital from 2007 to 2012. During this period, cerclage cable fixation was applied for acetabulum fractures with posterior column fractures without comminuted quadrilateral surface fractures. A patient underwent plate and screw fixation via an anterior approach, a patient underwent screw fixation for a posterior wall fracture, and two geriatric patients with a comminuted articular fracture underwent primary THA combined with bone grafting after 3 months of conservative treatment. Only anterior column and anterior wall fractures were treated conservatively. We excluded two patients who received cerclage cable fixation because following them for ten years was difficult. All participants provided informed consent indicating their voluntary participation in this study and their right to withdraw at any time, informed via a letter and brochure. This study was conducted in compliance with the principles of the Declaration of Helsinki. Ethical approval for establishing the registry was obtained from our institution (reference number 804).

### 2.2. Surgical Procedure

All procedures in this study were performed by a single hip specialist who has been conducting hip surgeries for >10 years. Patients underwent surgery under general anesthesia and were placed in the lateral decubitus position for the posterolateral approach. After the tensor fascia latae and gluteus maximus muscles were split, the posterior borders of the gluteus medius and vastus lateralis were identified. The vastus lateralis was released from the femur, and trochanteric osteotomy in the sagittal plane with a thickness of 1.5 cm was performed using a sagittal bone saw and an osteotome [10]. The piriformis tendon was dissected from the underlying bone and capsule and retracted posteriorly to identify the greater sciatic notch. The osteotomized fragment was rotated anteriorly, and the gluteus medius and minimus muscles were detached from the ilium to identify the anterior inferior iliac spine (AIIS). Long-handled, curved, and tipped surgical forceps were inserted from the AIIS into the greater sciatic notch. The single 2.0 mm Dall–Miles Cable (Stryker Howmedica, East Rutherford, NJ, USA) was grasped with forceps and extracted anteriorly. If the cable failed to remain on the posterior column fragment, the authors carved a groove with a 2.5 mm drill to hold the cable. The cable was gradually tightened around the AIIS while reducing the fragments on fluoroscopy. Posterior wall fractures were restored using fluoroscopy and fixed with cannulated cancellous screws. At the end of the surgery, all flip osteotomies were fixed using two cancellous screws.

### 2.3. Postoperative Rehabilitation

The patients were permitted to sit upright on a bed two days after surgery. Straight leg raise exercises were started seven days later. Active abduction and passive adduction exercises were avoided for four weeks to prevent dislocation of the trochanteric osteotomy. The patients were restricted to touch weight bearing (up to 10 kg) on crutches for six weeks, with weight bearing increasing progressively to their full weight after eight weeks.

### 2.4. Clinical Evaluation

The demographic data extracted for each patient included background factors (sex, age, body mass index, Charlson comorbidity index [11], and smoking status), injury factors, injury mechanisms, injury severity score (ISS), and surgical factors (operation time and intraoperative blood loss). The ISS is an anatomical scoring system that provides an overall score for patients with multiple injuries. The ISS was evaluated based on the abbreviated injury scale (AIS), an anatomical scoring system that characterizes the severity of injuries in various body regions. It is calculated as the sum of the squares of the highest AIS code (each ranging from 0 to 6) in the three most severely injured ISS body regions. Six body regions are included: the head or neck, face, chest, abdominal or pelvic contents, extremities or pelvic girdle, and external; scores range from 3 (least injured) to 75 (most injured) [12].

The fractures in this study were classified according to the Judet–Letournel classification using simple plain radiography, including the anteroposterior pelvis, obturator oblique, and iliac oblique views, as well as computed tomography (CT). The Judet classification is commonly used as an anatomic and radiographic description of acetabulum fracture patterns containing the anterior and posterior walls and columns. Standard plain radiographs were routinely obtained one month after surgery, every three months until the two-year follow-up, and annually after that. CT scans were usually performed one week after surgery and at the six-month follow-up. The quality of reduction was classified using Matta’s criteria on postoperative plain radiographs according to three groups: anatomical reduction (reduction loss < 1 mm), imperfect reduction (displacement < 3 mm), and poor reduction (displacement > 3 mm) [13]. Satisfactory reduction at the articular face was defined as a concentric gap of <2 mm and step-off on CT scans at the six-month follow-up [14]. We also assessed implant failure, femoral head avascular necrosis (AVN), and hip osteoarthritis. Magnetic resonance imaging was used to detect AVN of the femoral head in patients who experienced hip pain exacerbation that was not evident in radiography. Hip osteoarthritis was classified using the Tönnis classification [15]. The classification consists of three progressive degrees of degenerative changes to the hip: Grade 1 indicates minor joint space narrowing and subchondral sclerosis of the femoral head and/or acetabulum; Grade 2 indicates moderate joint space narrowing, small subchondral cysts of the femoral head and/or acetabulum, and moderate loss of femoral head sphericity; Grade 3 indicates severe joint space narrowing, large subchondral cysts, and severe femoral head deformity.

Objective postoperative hip functional outcomes were assessed using the Japanese Orthopedic Association (JOA) hip score one year after surgery and at the annual follow-up. The JOA hip score covers four categories and comprises a 100-point scale: pain (40 points), range of hip motion (20 points), ambulatory status (20 points), and activities of daily living (20 points) [16].

### 2.5. Statistical Analysis

Categorical data, such as success rates, were evaluated using Fisher’s exact test; continuous variables were analyzed using Welch’s *t*-test. Radiographic assessments were performed by two orthopedic trauma surgeons. We calculated intraclass correlation coefficients (continuous data) and the Kappa coefficient (categorical data) for interobserver reliability; they were 0.71 and 0.86, respectively. The significance level was set at *p* < 0.05. All statistical analyses were performed using EZR software (version 1.68, Jichi Medical University, Tochigi, Japan) [17].

## 3. Results

**Figure 1:**
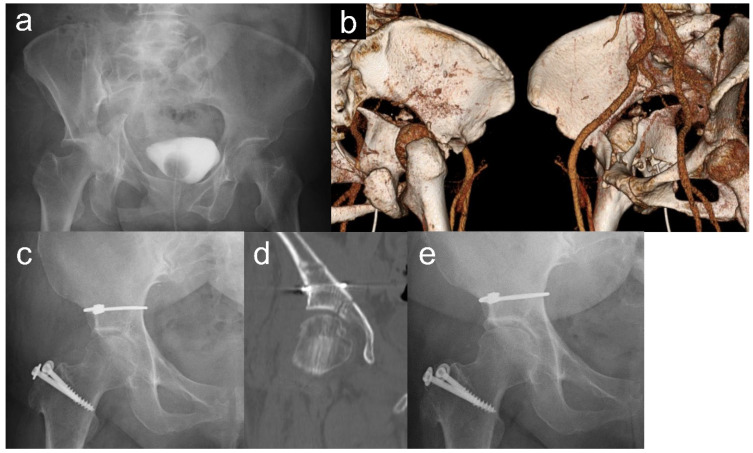
A case presentation of a 60-year-old woman injured due to a traffic accident. (**a**,**b**) Preoperative plain radiograph and computed tomography (CT) showed a T-shaped acetabular fracture. (**c**,**d**) Postoperative plain radiograph of cerclage cable fixation. The reduction quality is seen to be anatomical. (**e**) Plain radiograph at the 13-year follow-up. No osteoarthritis and no heterotopic ossification can be observed.

We identified nine patients who were treated for acetabular fractures and followed up for more than ten years. The patient demographics are presented in Table 1. The mean follow-up period was 14.1 ± 2.6 years (range: 10.8–18.1 years), and the mean age was 47.1 ± 15.5 years (range: 28–74 years). The mean ISS was 13.6 ± 4.7 (range: 9–22). Four patients were injured in traffic accidents, four were injured by falls from a height, and one was injured while snowboarding. Four cases were classified as both-column fractures, two were classified as T-shaped fractures, and two were classified as transverse posterior wall fractures. The representative case is shown in Figure 1.

**Figure 2:**
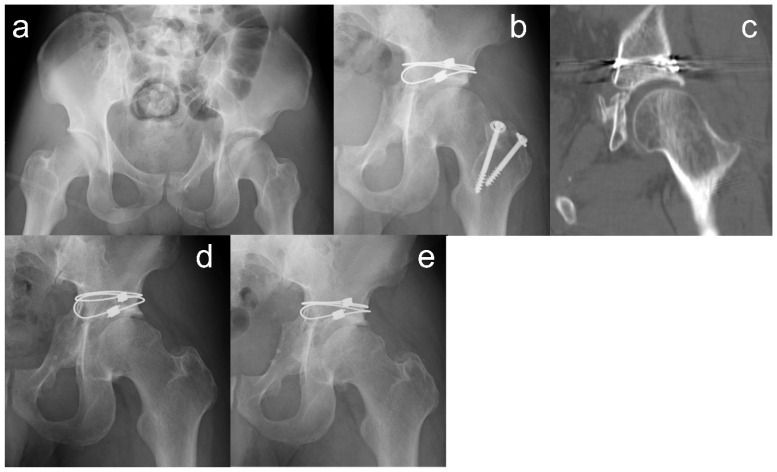
A case presentation of a 28-year-old man injured by a fall from a height. (**a**) Preoperative plain radiograph showed a dual-column acetabular fracture. (**b**,**c**) Plain radiograph and computed tomography at 6 months after surgery. The articular reduction quality is satisfactory and that of the anterior column is poor. (**d**) Plain radiograph at the ten-year follow-up. No osteoarthritis or heterotopic ossification were seen. (**e**) Plain radiograph at the last follow-up. The hip pain appeared approximately 12 years after surgery, and Tönnis grade I osteoarthritis was observed.

The mean waiting time from injury to surgery was 13.9 ± 7.0 days (range: 6–27 days). The mean intraoperative time was 199.1 ± 55.3 min (range: 143–330 min), and the mean blood loss was 667 ± 522 mL (range: 220–1941 mL). Anatomical reduction quality was achieved in five patients, as seen on plain radiographs, and satisfactory reduction of the articular surface was achieved in six patients, as seen on CT scans. Hip osteoarthritis was observed in four patients at the last follow-up (Table 2). One patient had Tönnis grade I osteoarthritis at the five-year follow-up, and his hip pain worsened approximately six years after surgery; radiographs showed Tönnis grade II hip osteoarthritis at the eight-year follow-up. One patient had no evidence of hip osteoarthritis until ten years postoperatively. However, hip pain appeared approximately 12 years after surgery, and radiographs at the last follow-up showed Tönnis grade I osteoarthritis (Figure 2). They were both under 40 years of age and engaged in physical labor. One patient aged 74 years with transverse column and posterior wall fractures developed AVN of the femoral head six months after surgery and Tönnis grade III osteoarthritis at the one-year follow-up. The mean JOA hip score was 88.1 ± 8.7 (range: 71–99) at the one-year follow-up and 90.9 ± 7.9 (range: 74–100) at the last follow-up. All patients were able to return to the same job they had before the injury, and the mean time from injury to their return to work was 20.2 ± 7.5 weeks. The detailed postoperative clinical outcomes for each patient are presented in Table 3.

## 4. Discussion

Cerclage cable fixation yielded satisfactory long-term functional and radiographic outcomes in this retrospective cohort study. The complex anatomy around the acetabulum and the relative inaccessibility of many types of acetabular fractures require an extensive approach, which is associated with the risks of HO, nerve disturbance, and intra-articular penetration by screws. Kang et al. reported that the cerclage cable technique for acetabular fractures showed good postoperative reduction quality and favorable functional outcomes after a midterm follow-up [9]. Park et al. indicated that cerclage cable fixation for displaced acetabular fractures obtained reasonably acceptable reductions in quality, functional outcomes, and soft tissue invasion [18]. This study showed that five of nine cases (55.9%) achieved anatomical reduction, as seen on plain radiographs; six of the nine patients (66.7%) achieved satisfactory articular reduction, as seen on CT scans. Previous studies showed an anatomical reduction rate of 71.2–73.9% [19,20] and a satisfactory articular reduction rate of 44.1–82.4% [21,22,23]. This reduction rate difference may be caused by the limited accessibility of the regions to restoration using the cable method.

The mean ISS in our study was 13.6 ± 4.7, indicating that many patients suffered multiple injuries, such as pulmonary contusion. Unlike fractures of the pelvic ring, acetabular fractures are unlikely to affect the difficulty of controlling bleeding in multiple traumas; therefore, early immobilization would be unnecessary. We could perform definitive fixation safely after a patient’s concomitant injuries have settled. Cerclage cable fixation enables the reduction and fixation of quadrilateral surface fragments using only a posterior approach, as shown in Figure 2. We consider this adaptation good for acetabular fractures requiring a posterior approach, such as those involving displaced posterior wall fractures. In contrast, some fracture types are difficult to treat with this method.

Cerclage cable fixation requires the posterior fracture line to extend into the greater sciatic notch. If the posterior fracture line is low, the cable is inserted through a drill hole created below the fracture line. However, reducing low anterior column and comminuted quadrilateral surface fractures that require an anterior approach is difficult. Therefore, plate fixation via an anterior approach might be better for fractures for which the anterior column fragment needs to be reduced or quadrilateral surface fragments are comminuted. We perform primary THA combined with impaction bone grafting for fragility acetabular fractures with displaced quadrilateral surface fragments caused by low-energy trauma.

In this study, three of nine patients (33.3%) showed postoperative osteoarthritis within two years after surgery; radiographic osteoarthritis increased to four of the nine patients (44.4%) in the subsequent ten years. Li et al. report that 70 of 217 patients (32.3%) who were treated for transverse acetabular fractures via the posterior approach developed postoperative osteoarthritis within two years of follow-up [20]. Briffa et al. report that the incidence rate of postoperative osteoarthritis was 68 out of 161 cases (38.5%) for a minimum of ten years after surgery [19]. Two patients with late hip osteoarthritis showed poor reduction quality on plain radiographs or unsatisfactory articular restoration on CT scans; their daily activity level was high. The imperfect reduction quality and high postoperative activity might be risk factors for late worsening of hip osteoarthritis; long-term follow-up would be necessary to detect late hip osteoarthritis for these patients. However, we did not observe any patients who underwent THA after a follow-up period of at least ten years. In a systematic review by Giannoudis et al., the THA revision rate after osteosynthesis for acetabular fractures was 8.5% [24]. One patient who had Tönnis type 3 osteoarthritis within two years of follow-up refused to undergo re-intervention for >10 years because she was able to perform daily activities despite experiencing pain. We hypothesized that older patients need less hip function in daily life and have a greater tolerance for hip pain.

We found that the JOA hip score was 90.9 ± 7.9 at the last follow-up; seven out of nine patients (77.8%) had a JOA score > 90. Briffa et al. reported that the proportion of patients with excellent or good functional scores using the modified Merle d’Aubigné and Postel scoring system was 72% (116 of 161 patients) [19]. Dodd et al. found that 70% (805/1146 patients) had “excellent” or “good” Harris hip scores [25]. Although these functional outcomes are not exactly comparable because the assessment items are different and the JOA score does not have a defined cut-off value, cerclage cable fixation showed favorable functional outcomes at long-term follow-up, according to previous studies.

The authors identified two patients with intraoperative bleeding of approximately 800 mL, and one patient bled approximately 2000 mL, although cerclage cable fixation is minimally invasive to the periacetabular soft tissues. Increased intraoperative hemorrhage was considered to result from damage to a branch of the superior gluteal artery around the greater sciatic notch during surgery and injury to an intramuscular blood vessel when a long clamp was placed anterior to the acetabulum. In particular, the superior gluteal artery can be injured by blind handling during clamp insertion from the anterior to the posterior. We indicated that protecting the vascular nerve bundle with the piriformis using a retractor is important to avoid such damage.

This study had some limitations. First, this study was retrospective with a small sample size. Retrospective studies have an inherent risk of observer and selection bias, including the potential for missing data and confounding variables. None of the patients in this study underwent THA during the follow-up. The rate of revision THA after osteosynthesis for an acetabular fracture was 8.5–10.5% in previous studies, and the most common reason is intra-articular hardware insertion [3,24]. If our sample size was larger, more patients would have undergone revision THA. Because acetabular fractures are rare, single-center studies have a limited sample size; a multicenter study would be necessary to increase the sample size. Second, this study was not comparative. Cerclage cable fixation showed satisfactory postoperative reduction quality and relatively good clinical results compared to previous studies on conventional osteosynthesis; however, our study could not determine the actual difference in clinical outcomes. Case-controlled comparative studies with matching fracture types comparing this method and plate fixation via a posterior or anterior approach with fracture type would be useful in the future. Two patients had worsening hip osteoarthritis > 5 years after surgery, but we could not evaluate the risk factors. Postoperative reduction quality and high activity were considered risk factors for its occurrence, but further research is needed to confirm this finding.

## 5. Conclusions

The cerclage cable fixation technique for acetabular fractures has shown good long-term functional outcomes and comparable postoperative complications. Long-term follow-up might be necessary for patients whose postoperative reduction is not anatomical, because even if no osteoarthritis is evident during short-term follow-up periods, late osteoarthritis may occur with longer follow-ups. We believe this method might be a good option for acetabular fractures with posterior column fractures. Due to the limitation of this study, namely having a small sample size and being a retrospective study with a risk of selection bias, further studies such as multicenter studies or prospective studies need to be conducted.

## Figures and Tables

**Table 1:** Patient demographics.

Number, *n*	9
Age, years, mean ± SD	47.1 ± 15.5
Sex, M/F, *n*	7/2
BMI, kg/m^2^, mean ± SD	24.8 ± 3.3
Smokers, *n*	4
Job before injury, *n*	
Farmer	1
Physical worker	3
Office worker	2
Injury mechanism, *n*	
Traffic accident	4
Fall from height (>2 m)	4
Snow sports injuries	1
ISS, mean ± SD	13.6 ± 4.7
Judet–Letournel classification, *n*	
BC	3
BC + PW	1
TV	2
TV + PW	1
TS	2
Acetabular roof fractures, *n*	3
Native hip dislocation, *n*	1

SD: standard deviation, BMI: body mass index, ISS: injury severity score, BC: both columns, PW: posterior wall, TV: transverse, TS: T shape.

**Table 2:** Postoperative complications and functional outcomes.

Number, *n*	9
Time from injury to surgery, days, mean ± SD	13.9 ± 7.0
Operation time, min, mean ± SD	199.1 ± 55.3
Blood loss, mL, mean ± SD	667 ± 522
Reduction quality on PR, *n* (%)	
Anatomical	5 (55.6)
Imperfect	3 (33.3)
Poor	1 (11.1)
Articular reduction quality on CT, *n* (%)	
Satisfactory	6 (66.7)
Unsatisfactory	3 (33.3)
Complications, *n* (%)	
DVT	1 (11.1)
Infection	1 (11.1)
Nonunion of trochanteric osteotomy	1 (11.1)
AVN of femoral head	1 (11.1)
Osteoarthritis	4 (44.4)
JOA at one-year follow-up, mean ± SD	88.1 ± 8.7
JOA at the final follow-up, mean ± SD	90.9 ± 7.9

SD: standard deviation, DVT: deep venous thrombosis, AVN: avascular necrosis, PR: plain radiograph, CT: computed tomography, JOA: Japanese Orthopedic Association hip score.

**Table 3:** Summary of patients.

Case	Age	Sex	Follow-Up	Mechanism	ISS	Time for Surgery	Fracture Type	Roof Fracture	Operation Time	Bleeding	Reduction Quality	Osteoarthritis	JOA at One Year	JOA at Last Follow-Up
			Years			Days			Minutes	mL				
1	38	M	18.1	Snowboard	9	11	BC + PW	−	209	309	Anatomical	Tönnis II	93	84
2	44	M	15.3	TA	10	17	TV	−	190	482	Anatomical	−	99	93
3	64	M	15.0	Fall	18	15	BC	−	155	574	Imperfect	Tönnis I	79	98
4	28	M	16.3	Fall	18	27	BC	−	330	1941	Poor	Tönnis I	87	91
5	39	M	14.6	Fall	22	11	BC	+	186	596	Anatomical	−	91	100
6	34	M	14.5	Fall	9	6	TV	+	222	801	Imperfect	−	93	93
7	61	F	10.9	TA	14	22	TS	−	194	800	Anatomical	−	85	90
8	74	F	11.2	TA	11	8	TV + PW	+	163	281	Imperfect	Tönnis III	71	74
9	42	M	10.8	TA	11	8	TS	−	143	220	Anatomical	−	95	95

ISS: injury severity score, JOA: Japanese Orthopedic Association hip score, TA: traffic accident, BC: both columns, and PW: posterior wall, TV: transverse, TS: T shape.

## Data Availability

The datasets used and/or analyzed during the current study are available from the corresponding author on reasonable request.
